# Transcriptomic Analysis of Wheat Under Multi LED Light Conditions

**DOI:** 10.3390/plants14010046

**Published:** 2024-12-27

**Authors:** Lei Sun, Ding Li, Chunhong Ma, Bo Jiao, Jiao Wang, Pu Zhao, Fushuang Dong, Shuo Zhou

**Affiliations:** 1 Hebei Key Laboratory of Plant Genetic Engineering, Institute of Biotechnology and Food Science, Hebei Academy of Agriculture and Forestry Sciences, Shijiazhuang 050051, China; 2 Dry-Land Farming Institute, Hebei Academy of Agricultural and Forestry Sciences, Hengshui 053000, China

**Keywords:** mono-light, transcriptome analysis, circadian clock

## Abstract

Light is a vital environmental cue that profoundly influences the development of plants. LED lighting offers significant advantages in controlled growth environments over fluorescent lighting. Under monochromatic blue LED light, wheat plants exhibited reduced stature, accelerated spike development, and a shortened flowering period with increased blue light intensity promoting an earlier heading date. In this study, we conducted a comprehensive transcriptome analysis to investigate the molecular mechanisms underlying wheat plants’ response to varying light conditions. We identified 34 types of transcription factors (TFs) and highlighted the dynamic changes of key families such as WRKY, AP2/ERF, MYB, bHLH, and NAC, which play crucial roles in light-induced gene regulation. Additionally, this study revealed differential effects of blue and red light on the expression levels of genes related to hormones such as cytokinin (CK) and salicylic acid (SA) synthesis as well as significant changes in pathways such as flavonoid biosynthesis, circadian rhythms, chlorophyll synthesis, and flowering. Particularly, blue light upregulated genes involved in chlorophyll synthesis, contrasting with the downregulation observed under red light. Furthermore, blue light enhanced the expression of anthocyanin synthesis-related genes, such as CHS, underscoring its role in promoting anthocyanin accumulation. These findings provide valuable insights into how light quality impacts crop growth and development.

## 1. Introduction

Given the increasing global population, resource scarcity, and environmental changes, traditional breeding methods are becoming insufficient to meet the growing demand for food. Modern biotechnological approaches and accelerated breeding strategies offer promising solutions to enhance wheat yield and quality [1]. Shortening the generational cycle of wheat through greenhouse cultivation using LED lights presents an effective strategy for accelerating the breeding process. One approach to enhance these conditions is by manipulating the light environment, including adjusting the light intensity and wavelength. A diverse selection of powerful LEDs suitable for use as a growth irradiance source in controlled environments became available at the beginning of the 20th century [2], which also enabled the investigation of light quality effects independently of the amount of photosynthetic irradiance.

Light is a fundamental environmental component that serves not only as the primary energy source for photosynthesis, but also as a crucial signal that guides plant growth and development throughout their entire life cycle. These developmental responses are regulated by various photoreceptors, such as phytochromes that are primarily sensitive to the red-to-far-red light ratio, blue light receptors, and photosynthetic pigments [3]. In Arabidopsis thaliana, over 13 photoreceptors have been identified, including phytochromes (PHYA-PHYE): red (R, 600–700 nm) and far-red (FR, 700–760 nm) light receptors that play key roles in plant photomorphogenesis and shade avoidance [4,5]; phototropins (PHOT1 and PHOT2): blue light (400–500 nm) receptors that mediate phototropism and stomatal opening [6]; cryptochromes (CRY1 and CRY2): blue/ultraviolet-A (UV-A, 320–400 nm) light receptors that influence circadian rhythms and photomorphogenesis [7]; and UVR8: a UV-B (280–320 nm) light photoreceptor that triggers UV-B-induced responses [8]. Recent studies highlighted the importance of specific wavebands, such as red/far-red and UV-A/blue in plant development. Research showed that red/far-red stimulates the conversion of phytochrome Pr (inactive form) to Pfr (active form), influencing plant height and photosynthetic capacity [9]. Blue light is more critical than red light for the function of the photosynthetic apparatus since blue light induces stomatal opening and contributes to a higher photosynthesis rate [10]. Green/yellow light (500–600 nm) has been shown to act as sensory systems that interact with blue and red sensors to regulate plant growth [11].

Photomorphogenesis encompasses multiple aspects of plant development, including plant architecture, flowering time, and other key processes such as seed germination, chlorophyll synthesis, etc. Monochromatic red light can reduce photosynthetic capacity and promote plant height elongation in cucumbers [12]. Studies have shown that plants tend to develop more tillers when exposed to a higher red-to-far-red light ratio [13] as branching determines the architecture of aboveground plant parts. Stem elongation and leaf expansion were reported to be linked closely to red and blue light [14] and root growth promotion was found in cabbage under red light [15]. A reduction in the red-to-far-red light (R/FR) ratio enhances the expression of the *CONSTANS (CO)* and florigen gene *FLOWERING LOCUS T (FT),* thereby promoting early flowering in Arabidopsis [16]. Light quality has also been shown to influence crop yield and quality, with red light impacting wheat growth and physiological indices [17]. Moreover, modifying light quality and quantity can optimize growth conditions and manipulate metabolism, yield, and quality in wheat [18]. There is an overall trend toward higher biomass production and photosynthetic capacity when blue light is included in the irradiance. The higher photosynthetic rates in wheat were associated with increased total nitrogen content in the leaves, which corresponded to greater amounts of key components involved in photosynthesis-limiting processes, including Rubisco, cytochrome f, chlorophyll, and major light-harvesting complex II (LHCII) [19].

The regulation of plant responses to light is a complex network that involves the interplay of hormones and transcription factors. The blue light-mediated alleviation of dormancy in Arabidopsis seeds was found to be related with gibberellin (GA) and abscisic acid (ABA) [20]. Blue light suppression alters cytokinin homeostasis in wheat leaves senescing under shading stress [21]. The contents of endogenous cytokinins were decreased in wheat seedlings grown under red light, especially when the shoot base was exposed. The application of exogenous cytokinin and its analog to the roots of seedlings grown in red light reversed the downregulation of the greening process [22]. An integrated transcriptome and metabolome analysis under varying light conditions demonstrated the involvement of multiple transcription factors, such as MYB and bHLH, in the plant’s response to light [23]. The bZIP-type transcription factor, ELONGATED HYPOCOTYL 5 (HY5), regulates gene expression during de-etiolation, and other bZIP transcription factors also participate in this regulatory process [24]. GATA transcription factors (TFs), which play roles in light response regulation, chlorophyll synthesis, and metabolism, have been found to be involved in the synthesis of terpenoid indole alkaloids in Uncaria rhynchophylla [25].

To fully understand the mechanisms of plant response to different monochromatic light sources, further research is needed. Although the effects and mechanisms of different light conditions on plants have been widely reported, there is limited information specifically on wheat. Moreover, the dynamic changes in gene expression in response to different light wavelength conditions over time have not been explored. Therefore, we propose a time course transcriptomic analysis of wheat under different colored light treatments with a focus on temporal dynamics to gain new insights into the regulation of wheat growth and development by light.

## 2. Results

### 2.1. Plant Growth and Development Under Blue Light

**Figure 1:**
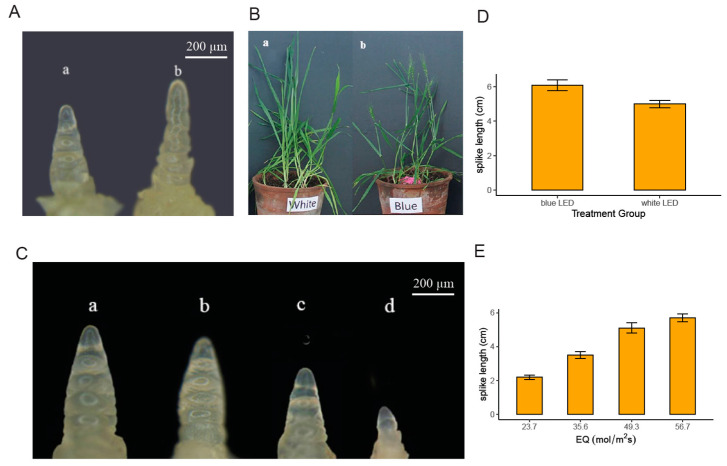
Effects of different light treatments on plant growth. (**A**) Development of spikes under blue light and white light treatments, (a) white light; (b) blue light; (**B**) height of the plants; and (**C**) development of spikes under varying light intensities, (a) 56.7 μmol/m^2^ s; (b) 49.3 μmol/m^2^ s; (c) 35.6 μmol/m^2^ s; and (d) 23.7 μmol/m^2^ s. (**D**,**E**) Statistics of spike length under blue and white light conditions and varying light intensities, respectively.

The developmental pace of wheat was assessed under blue light by monitoring the progression to young spike differentiation and the heading stage. Spike development proceeded rapidly under blue light, reaching the double-ridge stage, whereas it remained at the single-ridge stage under white light. The onset of the heading stage under blue light occurred 13.5 days earlier compared to the control (white light) (Figure 1A,B).

Both blue and white light treatments were adequate to support the growth and development of wheat. The biomass yield was higher under white light; however, the blue light treatment did not show a statistically significant difference in biomass yield when compared to white light (Table 1).

### 2.2. Effect of Light Intensity on Wheat Development Under Blue Light

After 20 days of planting, we selected plants from each light treatment group to examine the wheat spikes. The level of spike differentiation was observed to vary depending on the intensity of the light. Specifically, an increase in light intensity corresponded with a higher rate of spike differentiation. Across all the light treatments, the spikes were found to be at the single-edge stage; however, there were noticeable differences in the degree of spike differentiation between the various treatments (Figure 1C).

A positive correlation was noted between light intensity and the onset of the heading stage; as light intensity increased, the heading date occurred earlier. Specifically, plants exposed to a light intensity of 56.7 μmol/m^2^ s headed 3 to 4 days sooner than those under 23.7 μmol/m^2^ s. At the lower light intensity of 23.7 μmol/m^2^ s, the conditions were insufficient to support adequate wheat growth, resulting in a plant mortality rate exceeding 70%. In contrast, the other three higher light intensity treatments provided sufficient conditions to meet the basic requirements for wheat growth and development. Under blue light, the biomass yield was found to be influenced by light intensity, with higher intensities leading to greater biomass production. Thus, a moderate increase in light intensity can effectively shorten the growth cycle and enhance wheat yield (Table 2).

### 2.3. Expression Patterns of Wheat Genes Under Variable Light Conditions

The variable light environment impacts plant fitness by modulating the transcription of relevant genes. Considering that the expression of light-responsive genes changes rapidly after light exposure, we conducted a time course transcriptomic analysis to determine the gene expression patterns at 1 h and 6 h post treatment, as well as 14 days for a longer-term perspective, using different light qualities in vernalized wheat seedlings. For this analysis, three independent biological replicates were utilized to ensure the accuracy and reliability of the results. Four different light spectrums were employed. The photosynthetic photon flux density (PPFD) for the four light treatments was maintained consistently at 180 ± 5 μmol/m^2^ s, as detailed in (Table 3 and Table S1). Consequently, A total of 2.874 billion high-quality filtered reads were generated from 39 libraries, with Q30 percentages ranging from 95.9% to 97.6%, which further confirms the high quality of the sequence data. The clean reads were aligned to the reference genome of CS (IWGSC RefSeq v2.1 https://urgi.versailles.inrae.fr/download/iwgsc/IWGSC_RefSeq_Assemblies/v2.1/ (accessed on 17 May 2024)), achieving an average alignment rate of 95.19%, indicating that the sequencing data are reliable and adequate. The fundamental sequencing statistics are detailed in Table S2.

**Figure 2:**
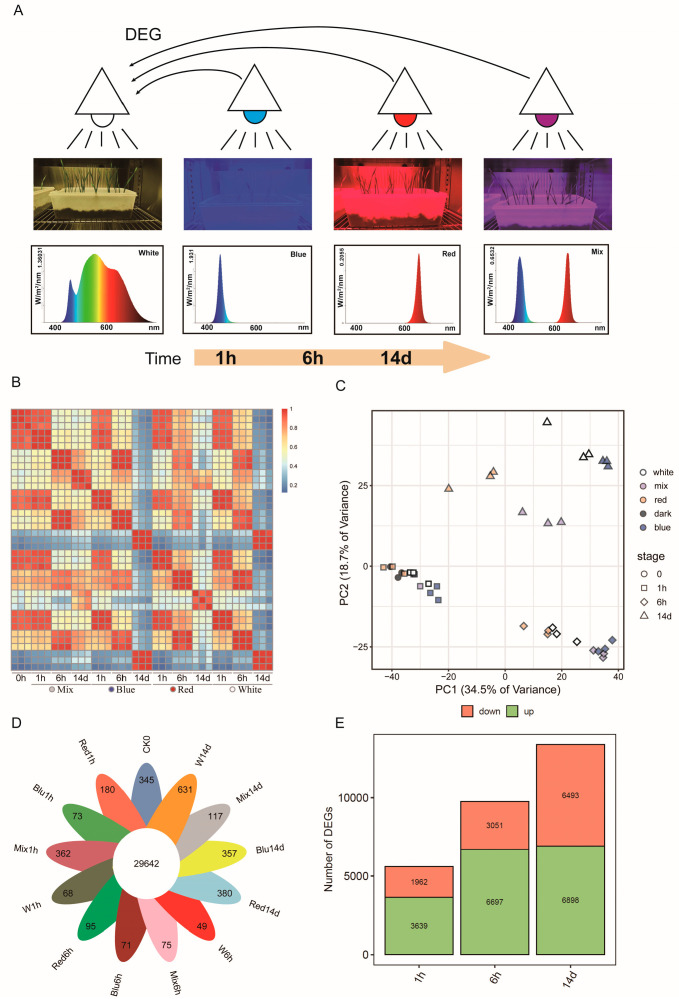
Global view of transcriptome expression and differential gene expression. (**A**) The experimental design schematic. The experiment included four light treatments: blue light, red light, white light, and a 1:1 mixture of red and blue light. Exposure was initiated from dark conditions, and samples were taken at three time points: 1 h (1 h), 6 h (6 h), and 14 days (14 d). Differentially expressed genes (DEGs) were identified by comparing the gene expression profiles at each time point under the respective light treatments with those under white light conditions. (**B**) Spearman correlation coefficient (SCC) of gene expression profiles between samples; (**C**) principal component analysis (PCA) of samples distinguished by different colors with three biological repeats; (**D**) petal plot, where each petal represents the number of uniquely expressed genes during that time period; and (**E**) number of differentially expressed genes over time under white light conditions.

The uniquely aligned reads from each sample were used to compute the normalized expression levels of each protein-coding gene quantified as fragments per kilobase of transcript per million mapped reads (FPKM). To eliminate genes with low expression levels, we summed the expression levels of genes across all samples and used a total sum greater than 1 as the screening criterion. A total of 67,179 expressed genes filtered from 107,891 mapped genes were identified. The Spearman correlation coefficients (SCC) between the three biological replicates ranged from 0.91 to 0.99, indicating a high level of consistency among replicates (Figure 2B). The PCA results show that the samples clustered into three groups according to the three sampling time points (i.e., 1 h, 6 h, and 14 d) (Figure 2C).

The petal plot shows, of all the expressed genes, 29,642 (44.12%) produced mRNAs detected in all samples (Figure 2D), whereas 37,537 genes (55.87%) were expressed only under specific light backgrounds and at particular sampling time points. The peak number of expressed genes occurred at the 14-day time point, while at the 6 h time point, no sample had more than 100 expressed genes.

### 2.4. Differential Gene Expressions and Transcription Factors Response to Light

Differentially expressed genes (DEGs) were identified using an adjusted *p*-value (padj) < 0.05 and an absolute log2 fold change (|log2FC|) > 2 as the thresholds. When compared to the dark environment control, 3639, 6697, and 6898 genes showed increased expression levels, while 1962, 3051, and 6493 genes exhibited decreased expression in response to white light treatment at 1 h, 6 h, and 14 d (Figure 2E). In comparison with white light treatment at the same time point (i.e., 1 h, 6 h, and 14 d), the corresponding number of up-regulated genes were 361, 72, and 4147 under red light; 739, 1370, and 1175 under blue light; 406, 578, and 2485 under red-to-blue 1:1 light, with that of 550, 230, and 2288 genes under red light; 624, 829, and 309 under blue light; and 1841, 632, and 2372 under red-to-blue 1:1 light were down-regulated, respectively. A Venn diagram showed common or uniquely regulated genes at each time point compared to white light (Figure 2A and Figure S1). By using the R package Masigpro (R package version 1.78.0.), the expression patterns of genes can be categorized into nine major classes (Figure S2).

Transcription factors (TFs) play critical roles in controlling plant growth, development, and phase transitions by regulating gene expression. To understand the response and metabolic changes in plants under different light spectra, DEGs in the comparison of three different light conditions with white light at the same time point were aligned against the iTAK database using HMMER models. A total of 34 different types of transcription factors were identified (Table S3). The five most abundant transcription factor types in sequence are WRKY, AP2/ERF, MYB, bHLH, and NAC.

**Figure 3:**
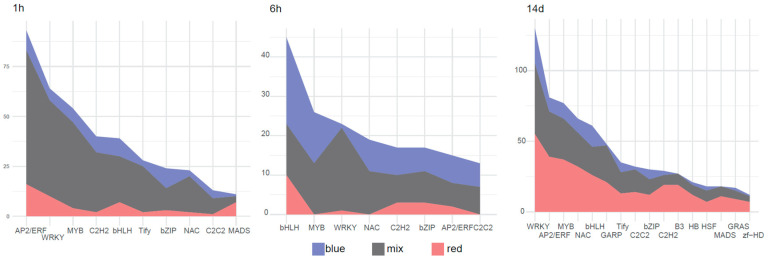
Transcription factor ridge plot showing the changes in number and types of transcription factors under different light conditions compared to white light at 1 h, 6 h, and 14 d.

In the short-term at both the 1 h and 6 h time points, the number of transcription factors among DEGs under mixed light conditions was more abundant than that under other light treatments. In contrast, during the long-term assessment period of 14 days, the number of TFs within the set of DEGs as well as the total number of DEGs under red light conditions were notably the highest (Table S3). This suggests that red light conditions showed the greatest disparity to normal light treatment in plant physiological regulation compared to other light sources over extended periods. To visualize the dynamic changes in TF abundance over time, we used a ridge plot (Figure 3), which displayed the fluctuation of different TFs under various light treatments. At the 1 h time point, the AP2/ERF family was the most numerous TF across all conditions, with AP2/ERF, WRKY, MYB, and bHLH being particularly abundant under mixed light conditions. By the 6 h mark, bHLH had become the most prevalent TF across all conditions. Interestingly, after 14 days of treatment, the number of bHLH TFs decreased to the fifth most abundant type, suggesting a shift in the regulatory mechanisms as the plants adapted to the prolonged light conditions. These observations highlight the dynamic nature of transcription factor involvement in plant responses to varying light conditions, indicating that different TFs are activated and participate in the regulation of gene expression at distinct temporal stages.

### 2.5. Expression of Hormone Pathway-Related Genes

**Figure 4:**
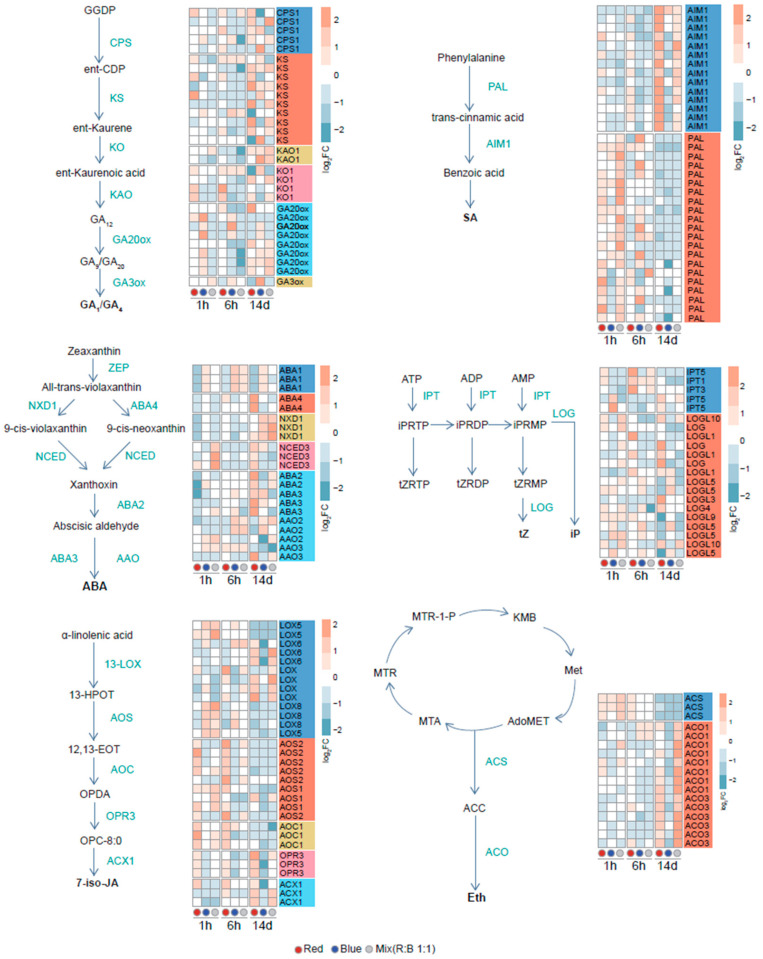
Expression changes in hormone synthesis-related genes under different light treatments. Heatmaps represent the log2 fold change (log2FC) values of gene expression levels involved in the GA, SA, ABA, CK, JA, and ethylene synthesis pathways compared to white light conditions. Each time point is represented by three treatments in three colors: blue for blue light, red for red light, and gray for a 1:1 mixture of blue and red light.

To better understand the effects of monochromatic light on endogenous hormone-related gene expression, we analyzed the transcriptional patterns of genes involved in phytohormone synthesis pathways in wheat under different light quality treatments (Figure 4). DEGs involved in hormone synthesis were listed and visualized through a heatmap. For gibberellin (GA) biosynthetic enzymes, we observed genes encoding GA3 oxidase (GA3OX) and GA20 oxidase (GA20ox) were upregulated under blue light conditions. For abscisic acid (ABA), the expression levels of genes encoding ABA1, which is a key enzyme in the ABA biosynthesis pathway, were consistently decreased under red light and increased under blue light across all time points examined. Another hormone of interest, ethylene, was also studied through the analysis of ACC synthase (ACS) and ACC oxidase (ACO) genes. ACS showed an initial increase in expression levels shortly after the onset of different light treatments, indicating a potential rapid response to light changes. However, after 14 days of treatment, the expression levels of ACS genes generally declined, suggesting a shift in ethylene biosynthesis dynamics over time. Specifically, at the 14-day time point, the expression levels of the ACO gene family decreased under blue light treatment alone; however, a large portion of ACO and ETO1-related genes showed increased expression under red light and under a 1:1 mixture of red and blue light conditions, implying a unique regulatory mechanism for ethylene production under blue light conditions. For the salicylic acid (SA) synthesis pathway, it was observed that AIM1 was significantly upregulated by red light after a 14-day exposure. On the other hand, the expression of phenylalanine ammonia-lyase (PAL), an enzyme involved in SA synthesis, was prominently induced by red light in the short term. After 14 days, however, multiple genes associated with PAL synthesis were notably downregulated under blue light. Among the LOG family genes, which play a role in cytokinin (CK) biosynthesis, most showed reduced expression levels under red light compared to blue and white light at the 14-day mark. Significantly, the expression levels of LOG9 and LOG5 were markedly decreased under red light conditions. These findings indicate that different wavelengths of light can have distinct impacts on the expression of genes involved in phytohormone synthesis pathways, thereby influencing the hormonal balance within the plant. Our transcriptomic analysis reveals differences in the expression of genes involved in hormone synthesis pathways under different light conditions, suggesting a possible link to variations in plant growth and development.

In the expression of genes related to plant hormone signaling pathways, the number of differentially expressed genes under three light conditions (blue, mixed, and red light) compared to white light showed that blue light has the fewest changes in gene expression, with the overall number of differences being the least compared to red light and mixed light. Over time, the changes were most pronounced at early (1 h) and late (14 days) time points, with fewer changes at 6 h. This indicated that the plant hormone signaling pathways exhibited rapid fluctuations shortly after transitioning from darkness to light, then stabilized after a period of time, and ultimately showed significant differences from white light after prolonged exposure to different light conditions at 14 days (Figure S3).

### 2.6. Functional Enrichment Analysis

**Figure 5:**
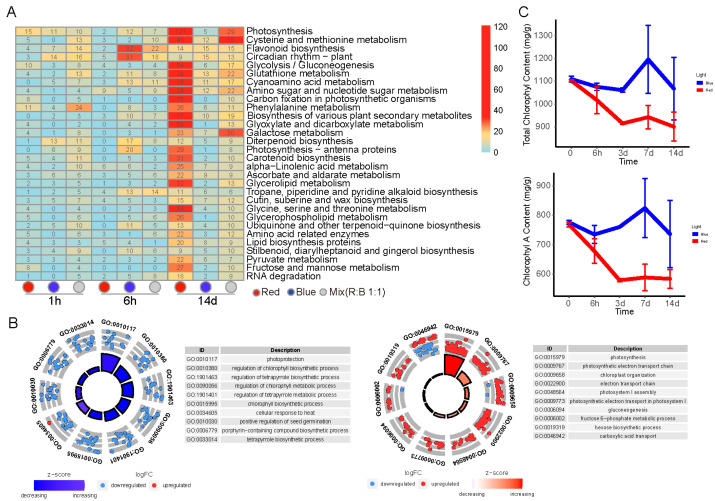
Analysis of pathway enrichment in response to different light conditions. (**A**) KEGG enrichment analysis. KEGG pathway enrichment analysis was performed on differentially expressed genes (DEGs) identified by comparing the gene expression profiles under each light treatment with those under white light conditions at the same time points. The pathways were ranked according to the total number of enriched genes across all conditions, and the results are visualized using a heatmap, with the specific differentially expressed genes and their enrichment analysis under various light conditions relative to white light. (**B**) GO enrichment analysis of unique DEGs. By comparing samples from each light condition with those from white light, genes that were significantly differentially expressed under each light condition were filtered, then unique condition-specific differential DEGs were screened for GO enrichment analysis. Left and right panels are GO enrichment analysis of unique DEGs in white light compared to blue and red light at 14 d, separately. (**C**) Changes in chlorophyll content after treatment under blue and red light.

KEGG enrichment analysis was conducted using the local KofamKOALA database. DEGs were enriched in some metabolism-associated pathways such as photosynthesis cysteine and methionine metabolism, flavonoid biosynthesis, glutathione metabolism, and cyanoamino acid metabolism (Figure 5A). Flavonoid biosynthesis and circadian rhythm-related genes were found most under 6 h blue light. Our study found that after blue light exposure, plants showed earlier enrichment of DEGs in certain metabolic pathways compared to red light exposure. Within short periods (1 h and 6 h), blue light exposure resulted in a significant enrichment associated with the diterpenoid biosynthesis and cyan amino acid metabolism pathways. In contrast, at the same time points, red light exposure led to fewer DEGs being enriched in these pathways when compared to white light exposure. However, under prolonged exposure conditions (14 d), red light exposure resulted in a substantial enrichment of differentially expressed genes within these metabolic pathways. Additionally, the fructose and mannose metabolism pathways were particularly evident among the DEGs under red light exposure.

To clarify the specific differences induced by each light treatment, the GO enrichment analyses were further conducted based on DEGs, which were unique to each treatment at the same time point with white light as control (Figure 5B and Figure S4). Chlorophyll-related pathways were found upregulated under blue light. In contrast, chlorophyll-related pathways were downregulated under red light. Some stress-related pathways such as osmotic stress and salt stress appeared at 1 h under the mixed light condition. To verify the changes in the expression of chlorophyll synthesis-related genes under different light conditions, we measured chlorophyll content in plants exposed to blue and red light over a period of 14 days (Figure 5C). Chlorophyll content was measured at four time points: the 1st, 3rd, 7th, and 14th days. All measurements were taken during the same time period each day, and each treatment was performed in duplicate at each time point. The results show that total chlorophyll and chlorophyll a content were consistently higher under blue light compared to red light throughout the experimental period, with a notable peak observed on the seventh day. In comparison to the zero-time control samples (dark treatment after vernalization under white light), chlorophyll content was lower under red light, indicating that chlorophyll synthesis under red light conditions was less efficient compared to blue light.

### 2.7. Genes Related to Circadian Clock

**Figure 6:**
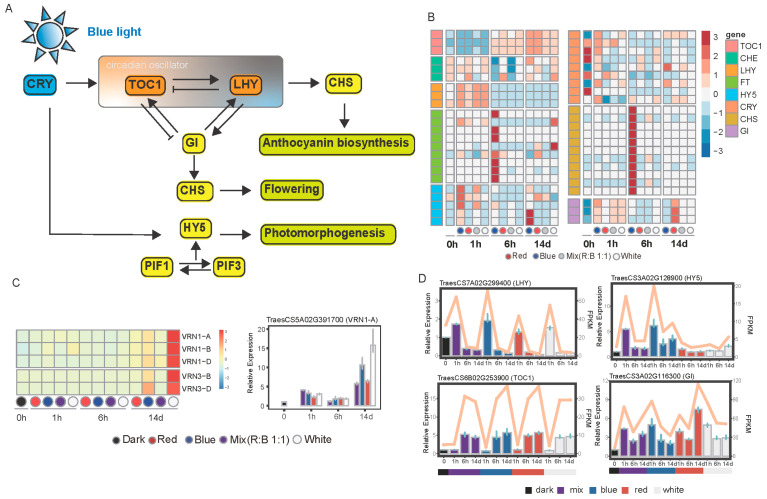
Genes associated with the circadian clock. (**A**) Partial and core genes in the circadian rhythm plant pathway. (**B**) Heatmap showing the expression levels of clock-related genes. (**C**) Heatmap illustrating the expression levels of VRN family genes, supplemented with qRT-PCR validation results. (**D**) qRT-PCR validation and RNA-seq expression profiles for selected genes.

Combined with earlier studies and based on our KEGG pathway enrichment analysis results, we found that different light sources have substantial effects on circadian rhythms. Therefore, we analyzed the expression levels of genes in the relevant pathways (Figure 6). We found the expression of TOC1 was upregulated as time advanced. In contrast, LHY showed the opposite pattern, consistent with the feedback loop. According to the KEGG database, the circadian rhythm pathway influences the plant photomorphogenesis, flowering, and UV-B protection. It is noteworthy that the key genes involved in these pathways displayed distinct expression patterns under blue light compared to other light conditions. The expression levels of FT and CHS were particularly high under blue light at 6 h, and HY5 at 1 h. VRN3 plays a crucial role in the vernalization pathway in wheat, contributing to the regulation of flowering time, which is also known one of the orthologues of FT. So, we also checked the expression level of genes in the vernalization pathway. The peak expression level of VRN1 and VRN3 was 14 d under white light, then the blue light, indicating that different light conditions have distinct effects on vernalization and flowering (Figure 6C). Four genes participating the circadian pathway were used to verify the accuracy of the transcriptomes by qRT-PCR, which showed consistency with the RNA-seq findings (Figure 6D).

### 2.8. Co-Expression Network Analysis of the Transcriptome

Weighted gene co-expression network analysis (WGCNA) is a robust systems biology technique that identifies and analyzes clusters of co-expressed genes, referred to as modules, from large-scale gene expression data. A total of 31,797 genes (covering 47.3% of the total expressed genes) identified to be DEGs between adjacent time points as well as across different light treatments compared to white light at the same time point were set as the input. We assessed the correspondence between the correlation coefficient and mean connectivity at various thresholds (power values ranging from 1 to 30) (Figure S4A). As illustrated in the figure, the optimal power value was set to 16. Additionally, 36 co-expressed gene modules were generated by WGCNA analysis (Figure S4B). The module–sample relationship was conducted based on the correlationship. “MEdarkgreen” showed a significant positive correlation with blue light at 6 h, while the “MEslamon” and “MEyellow” module had a significant positive correlation with red light at 1 h and 14 d, respectively. The GO enrichment analyses were performed for each module. Samples treated under each light source for 1 h were positively correlated with the “Mepink” module. GO analysis revealed that this module is associated with nucleic acid–protein assembly, light response, and circadian rhythms (Table S4). “Memagenta” modules were found to be positively associated with samples under white, red, and mix light conditions for 6 h, but not for blue light. This module is associated to photosynthesis-related pathways and certain lipid processes. This module may help explain the differences in morphogenesis between blue light and other light sources.

**Figure 7:**
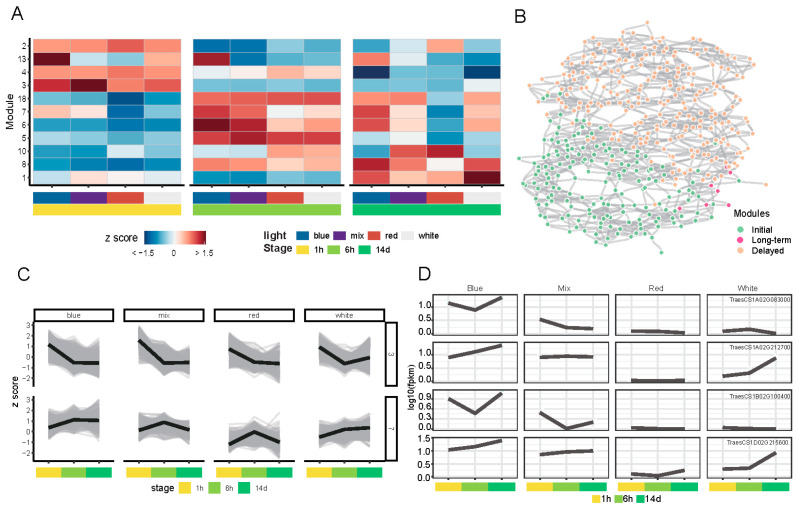
Gene co-expression network analysis. (**A**) Gene module classification heatmap showing the standardized log(fpkm) values of genes, which can be categorized into three classes based on their expression patterns. (**B**) Schematic diagram of selected gene network interactions. (**C**) Expression patterns of modules 3 and 7. (**D**) Expression patterns of four bHLH genes among 404 neighbor genes.

To further investigate the network patterns of gene co-expression, we employed the Simple Tidy GeneCox pipeline for co-expression analysis of the transcriptome. It seemed that HY5 and CHS represent two distinct expression patterns in response to varying light stimuli. Thus, these two genes were further selected as baits. To ensure appropriate scaling and centering of the expression data, transcriptome data were subjected to log transformation of FPKM values, followed by Z-score normalization. Based on the ranking of variance in mean logFPKM across all samples, the top 8000 genes, which constituted the upper elbow of the ranking plot, were identified and selected as high-variance genes (Figure S5). Based on the correlations between genes, Pearson correlation coefficients were calculated, and corresponding *p*-values and false discovery rates (FDR) were determined. To optimize the network structure, two criteria were prioritized: 1. maximizing the number of modules containing at least five genes; 2. maximizing the number of genes included in modules that contain at least five genes. For this purpose, a resolution parameter of 2 was selected based on the findings presented in (Figure S5), which facilitated the optimization of module sizes and gene distribution within the network. A total of 11 modules, each containing more than 5 genes, were identified (Figure 7A). Based on the timing of the peak expression levels, these modules can be categorized into three groups. The first is early rapid response modules (Module 2, 3, 4, and 13): genes that respond quickly to the stimulus. Second, intermediate response modules (Module 5, 6, 7, 8, 10, and 18): genes that show a delayed response. Lastly, long-term response module (Module 1): genes with sustained expression changes over a longer period.

Notably, HY5 was assigned to Module 2, classifying it as an early rapid response gene. In contrast, CHS was allocated to Module 7, with its expression peaking around 6 h post stimulation. The expression patterns of these two modules are illustrated in Figure 7C. A total of 404 neighbor genes were identified based on their correlations with HY5 and CHS. The top five edges with the highest absolute correlation coefficients (|r|) were selected from these genes and were subsequently used to construct a sub-network depicted in (Figure 7B). Gene ontology (GO) enrichment analysis of the 404 identified genes demonstrated their significant involvement in pathways related to various light stimuli and chlorophyll biosynthesis (Figure S5, Table S6). Additionally, these genes were enriched in various cellular compartments, including plastoglobules within the chloroplast membrane and peroxisomal membranes. This suggests a potential role of these genes in multiple aspects of plant photomorphogenesis and metabolic processes. Among the 404 neighbor genes, 18 genes were annotated as TFs: 3 AP2/ERF, 4 bHLH 3 C2C2, 2 WRKY, and 5 BHLH, with annotated homologous information provided in (Table S6). The bHLH transcription factor was found to be PIF3 and its homologous genes and was found to be induced by blue light exposure (Figure 7D).

## 3. Discussion

LED lighting offers significant advantages over fluorescent lighting in controlled growth environments. When LED lighting is used instead of fluorescent lighting, it has been shown to increase biomass by 40% and yield by 60%, while reducing energy consumption by 55% in wheat [18]. As a result, there has been a growing emphasis on understanding how light quality affects vegetable crops, with the aim of improving both the yield and quality growth. Our research demonstrates that under blue light LED treatment, wheat plants exhibit reduced stature, accompanied by accelerated spike development and a shortened flowering period. These findings align with the established role of blue light in promoting flowering [26,27]. Additionally, the intensity of light also significantly influences the growth and development of plants. Previous study on Gynura (Begonia fimbristipulata) has shown that different light intensities can lead to distinct growth and developmental outcomes. In our study, under controlled monochromatic blue light conditions with different intensities, we observed variations in the growth and development of wheat plants, including changes in spike emergence, survival rates, and dry weight.

RNA-seq technology offers significant advantages for analyzing transcriptome dynamics. In this study, a comprehensive transcriptome analysis was conducted to investigate the molecular mechanisms underlying the response of wheat plants to different light conditions over time. The high alignment rates and quality metrics indicate the reliability of the sequencing data. The identification of 67,179 expressed genes from a total of 107,891 mapped genes highlights the comprehensive coverage of the transcriptome. Our analysis of transcriptomes from vernalized wheat seedlings revealed a large number of differentially expressed genes (DEGs) across the developing course, indicating dynamic and complex gene regulation under different light conditions.

The identification of 34 different types of transcription factors (TFs) and their dynamic changes under different light treatments highlights the role of these regulators in mediating plant responses to light. The abundance of TFs, particularly WRKY, AP2/ERF, MYB, bHLH, and NAC, suggests that these families play crucial roles in light-induced gene regulation. The results are consistent with the fact that WRKY transcription factors (TFs) play a role in modulating flowering time in Arachis species through pathways related to aging, autonomous control, circadian rhythms, hormone signaling, photoperiod, sugar sensing, temperature responsiveness, and vernalization [28]. Ethylene response factors (AP2/ERF) have been shown to play a role in plant developmental processes and to regulate stress tolerance. Transcriptome and metabolome analyses revealed that genes associated with the photosynthesis pathway were significantly altered in the ERF10-KO mutant with a dwarf phenotype. This was accompanied by changes in the expression of bHLH, NAC, MYB, and WRKY transcription factors [29]. MYB transcription factor is one of the largest families in plants corresponding to abiotic stress [30]. The core component of circadian clock LATE ELONGATED HYPOCOTYL (LHY) and CIRCADIAN CLOCK-ASSOCIATED 1 (CCA1) are MYB TFs which negatively regulate TOC1 expression [31], and the expressions of both showed a significance change under various light conditions. CRY1 prevents the COP1-mediated degradation of BIT1, a MYB transcription factor, thereby activating blue light-dependent gene expression in Arabidopsis [32]. Analysis of DEGs under red and blue light treatments compared with white light controls revealed significant changes in several transcription factor families. Among these, we focused on transcription factors that exhibited markedly distinct expression patterns under different light conditions. Specifically, we identified three members of the basic leucine zipper (bZIP) transcription factor family that displayed opposite expression trends under red and blue light treatments. These bZIP transcription factors showed low expression levels under red light, but significantly increased expression levels under blue light. This finding suggests that these specific bZIP transcription factors may play a crucial role in plant responses to different light qualities.

We observed differences in the expression levels of genes related to hormone biosynthesis pathways under varying light treatments. Specifically, the expression level of gibberellin (GA) biosynthetic enzymes GA3OX and GA20ox was upregulated under blue light, and transcription factors involved in the GA biosynthesis pathway were enriched in the differentially expressed genes under blue light conditions. This suggests a significant relationship between blue light and GA, which aligns with previous reports indicating that GA promotes flowering [33]. Conversely, the expression levels of genes involved in abscisic acid (ABA) biosynthesis pathways were lower under blue light compared to white light, indicating an antagonistic effect of ABA on flowering [34]. Additionally, we noted significant differences in the expression of genes related to salicylic acid (SA) biosynthesis under red and blue light. Early on, the expression levels of phenylalanine ammonia-lyase (PAL), a key enzyme in SA biosynthesis, were similar or higher under both red and blue light compared to white light. However, after 14 days, the expression levels of PAL were lower under both red and blue light conditions. For the AIM1 synthase, the expression level was higher under red light, but lower under blue light compared to white light. These findings suggest that SA plays a critical role in regulating plant growth under different light conditions, as SA accelerates leaf senescence [35]. A previous study on broccoli showed a negative correlation between blue light and SA synthesis [36,37], which was consistent with our result. The differential effects of blue and red light on plant growth and flowering can also be reflected in the expression levels of genes related to cytokinin (CK) synthesis. Specifically, most of the LOG family genes, which are involved in CK biosynthesis, show lower expression levels under red light compared to blue and white light at the 14-day time point. Notably, the expression levels of LOG9 and LOG5 are significantly reduced under red light. It is worth mentioning that although we observed changes in the expression levels of hormone synthesis-related genes after log2 fold change transformation, the majority of these changes were not statistically significant. This suggests the intricate interplay between light and the complexity of hormonal regulation in plants.

The concept of pathway enrichment is a powerful tool in transcriptomic analysis, providing valuable insights into the molecular mechanisms underlying various biological processes. In our study, we employed pathway enrichment analyses based on the Kyoto Encyclopedia of Genes and Genomes (KEGG) and Gene Ontology (GO) to investigate the effects of different light conditions on plant pathways. Additionally, weighted gene co-expression network analysis (WGCNA) was used to identify distinct modules of co-expressed genes under varying light treatments. Our findings revealed that different light conditions significantly influenced key pathways in plants, including flavonoid biosynthesis, circadian rhythms, chlorophyll synthesis, and flowering. Specifically, under blue light exposure, genes involved in chlorophyll synthesis were upregulated, whereas they were downregulated under red light conditions. These results are consistent with prior research [38,39]. Plants grown under monochromatic red light suffer a spectral “deficiency” syndrome resulting from abnormal photosynthetic functioning and can be reversed by adding a small fraction of blue light [39]. Under blue light illumination, the expression pattern of the photoreceptor CRY1 shows significantly different changes compared to treatment with red monochromatic light. Specifically, at the 6 h time point, the expression level of CRY1 reaches its lowest under blue light; after 14 days, its expression is significantly higher than under other light conditions, including a 1:1 mixture of blue and red light. In contrast, the expression pattern of CRY2 remains largely unchanged across all light treatments, consistent with literature reports indicating that although CRY2 is involved in regulating plant development under blue light, its own expression level does not change but rather is regulated at the protein level [40]. These findings suggest that blue light affects photomorphogenesis through CRY2, and the regulatory process is complex, involving multiple layers of control at both the transcriptional and protein levels (Figure S6). All of these observations indicate that blue light promotes chlorophyll synthesis, while red light has an inhibitory effect.

The heading stage is a critical transition period for wheat growth and significantly influences yield. Photoperiod and light quality can influence the timing of flowering [41]. Our data show that wheat transitions to the reproductive stage earlier in blue light-treated wheat, which is consistent with previous reports indicating that blue light at different peak wavelengths (405, 450, and 470 nm) promotes early flowering compared to red light (peak wavelengths at 630 and 660 nm) in everbearing strawberry plants [42]. Notably, blue light significantly induces the light-regulated transcription factor HY5, which plays a key role in plant growth and development, including the integration of light and hormonal signaling pathways, regulation of flowering, and promotion of anthocyanin and flavonoid biosynthesis [43]. The rapid response of HY5 to blue light (upregulated after 1 h) indicates its role as a primary target of light signaling and its involvement in the immediate response to light. FT acts as one of the two homologous proteins controlling flowering transition and inflorescence architecture [44,45]. Likewise, the delayed upregulation of FT (after 6 h) suggests that it may be downstream of HY5 or part of a secondary pathway that requires additional signals or time for activation. Given that the homolog of the flowering gene FT is also a vernalization gene, VRN3 in wheat, this prompted us to further explore the expression levels of vernalization genes under different light conditions. Interestingly, at day 14, we observed higher expression levels of VRN1 under blue light compared to red light; however, the highest expression was not under monochromatic light, but under white light. This finding suggests that the induction of VRN genes likely requires the simultaneous action of multiple spectral components.

Anthocyanins are important secondary metabolites in plants, imparting vibrant colors to tissues such as petals, fruits, and leaves, and possessing antioxidant and other bioactive properties [46]. The effects of monochromatic light, particularly blue and red light, on anthocyanin synthesis have been extensively studied. One of the research findings indicates that supplementing with monochromatic blue LED light enhances the accumulation of anthocyanins in the berry skin of grapevines [47]. Our transcriptomic analysis reveals that the expression levels of chalcone synthase (CHS), a key gene involved in anthocyanin synthesis, are differentially regulated under different light treatments [48]. The transcriptional level changes of CHS are similar to those of FT, showing significant upregulation under blue light at 6 h. This is consistent with previous reports showing that blue light can induce the expression of a series of genes involved in anthocyanin biosynthesis including CHS [23]. However, CHS does not exhibit significant differences in expression levels under short-term (1 h) or long-term (14 d) exposure compared to other light sources. This suggests that the upregulation of FT and CHS requires a certain duration of induction and that their expression levels stabilize over time without being specifically enhanced by blue light under steady-state conditions. As a core regulator of anthocyanin content, HY5 induces the expression of CHI [49], which in turn regulates anthocyanin levels [50]. In this study, we observed that the expression of HY5 is induced by blue light, which suggests an upregulation of anthocyanin synthesis stimulated by blue light. Phytochrome-interacting factors (PIFs) are a family of bHLH transcription factors that plays critical roles in regulating light signaling pathways and various aspects of plant growth and development. Early research indicated that both PIF1 and PIF3 exhibit responsiveness to blue light, highlighting their significance in the blue light signaling pathway [51]. In this study, we employed a co-expression network analysis to explore the expression patterns of PIF1 and PIF3 alongside other genes. Our transcriptomic data reveal that the expression patterns of HY5 and PIF3 are highly similar, suggesting a potential co-regulation or interaction between these two genes. This finding is consistent with previous studies that identified a complex involving PIF1/PIF3, HY5, and BBX23, which plays a crucial role in light-regulated gene expression [52]. These results suggest that PIF3 and HY5 play an integrated role in the light signaling network, potentially forming part of a larger regulatory complex.

## 4. Materials and Methods

### 4.1. Plant Growth Conditions and Light Treatments

Winter wheat Jinghe9123 (Triticum aestivum) was obtained from our laboratory, and uniform seeds were germinated in incubators with wet vermiculite for 3 d at 25 °C. The 3-day-old plantlets were subjected to vernalization for 25 d at 6 °C under a 16/8 h light/dark photoperiod.

Monochromatic light treatments, including blue (peak wavelength, λmax = 460 nm) and red (λmax = 660 nm) lights along with white light, were administered using LED equipment. A light-emitting diode (LEDa) was installed in an incubator of a 1.3 m length, 0.45 m width, and 1.5 m height. The light quality parameters were estimated using a hand-held plant lighting analyzer (PLA-20; Yuan Fang Telecom Co., Ltd., Yixing City, China).

For long-term blue light treatment, plantlets of uniform size were grown in pots (diameter, 20 cm; height, 25 cm) filled with a 1:1:1 (*v*/*v*/*v*) mixture of garden soil, humus, and vermiculite. Nine pots of eight wheat seedlings each were placed in blue and white light conditions. The pots were placed randomly in the growth chambers. The plants were grown at 22 ± 3 °C under a 16/8 h light/dark photoperiod and were watered daily with tap water until maturity. For time course transcriptome analysis, seedlings were placed under dark adaptation overnight for 8 h and then exposed to red, blue, red and blue (ratio 1:1), and white light. Leaf samples were collected after 1 h, 6 h, and 14 days of each light treatment, each with 3 biological repeats. Prior to the light treatment, leaf samples were also collected in a dark environment to serve as the control group (0 h of light exposure).

### 4.2. Determination of Plant Growth and Development

Wheat plants were randomly selected after 25 d of planting under each light treatment, and spike differentiation was observed under a stereomicroscope (SZX16; Olympus, Tokyo, Japan). Heading time (when half of the ear was exposed) was recorded to further determine the wheat development rates under different light treatments.

At harvest, the total biomass of the plants, including the weight of the straw, leaves, and spikes, was determined for at least 20 randomly selected plants under each light treatment. The plants were harvested and dried in an oven for 48 h at 50 °C before weighing.

Total chlorophyll content was determined using the ethanol extraction method. Fresh leaf tissue (0.1 g) was homogenized in 5 mL of or 95% ethanol, incubated in the dark at room temperature for 24 h, and then centrifuged at 4000 rpm for 10 min. The supernatant was used for spectrophotometric analysis, with absorbance measured at 649 nm and 665 nm against distilled water as the blank. Chlorophyll a and b concentrations were calculated using the following equations:

Chlorophyll a (mg/L) = 13.95 × A_665_ − 6.88 × A_649_

Chlorophyll b (mg/L) = 24.96 × A_649_ − 7.32 × A_665_

Chlorophyll total (mg/g) = Chlorophyll total (mg/L) × V × N/W × 0.001.

### 4.3. RNA Extraction, Library Construction, and Sequencing

A total of 39 leaf tissue samples were used for sequencing. These samples were individually ground in separate RNase-free mortars filled with liquid nitrogen. Total RNA was extracted using the RNAprep Pure Plant Kit (Tiangen, Beijing, China) following the manufacturer’s instructions. The integrity of the RNA was assessed using the RNA Nano 6000 Assay Kit on the Bioanalyzer 2100 system (Agilent Technologies, Santa Clara, CA, USA). Subsequently, mRNA was isolated using VAHTS mRNA Capture Beads (Vazyme Biotech, Nanjing, China) according to the protocol. To generate the sequencing libraries, the NEBNext^®^ UltraTM RNA Library Prep Kit (NEB, Ipswich, MA, USA) was utilized. After PCR product purification and library quality assessment, RNA sequencing was performed on the Illumina Novaseq platform (Illumina, San Diego, CA, USA) by Personalbio (Shanghai, China). Sequenced raw reads in FASTQ format were filtered with FASTP v0.23 [53] to remove low-quality reads and reads containing adapters. The paired-end clean reads were subsequently mapped to the reference genome IWGCS (available at: https://www.wheatgenome.org/about/iwgsc-2.0 (accessed on 12 February 2024)) [54] using HISAT2 v2.2.1 [55] for alignment. The number of reads in each gene model, as shown in the GFF file, was counted using the R package FeatureCounts v1.5.0 [56], and each gene expression level was normalized as fragments per kilobase of transcript per million mapped reads (FPKM). To filter out low-expression genes, a total sum of FPKM values across all samples > 1 was applied. The results of principal component analysis (PCA) using the R package PCAtools v2.8. 0 [57] and correlation analysis based on the Spearman correlation coefficient method were used for quality analysis. Heatmaps were drawn using R package tidyheatmap [58] and bar plots were drawn using the R package ggplot2 [59].

### 4.4. Differential Expression, Functional Annotation, and Co-Expression Network Analysis

Differentially expressed genes (DEGs) were identified using the R package DESeq2 v1.20. 0 [60]. The *p*-value was adjusted using the Benjamini and Hochberg method, and a *p*-value (padj) < 0.05 together with an absolute log2 fold change > 2 were used as the cutoff criteria for screening significant DEGs. The analysis of common and unique DEGs between different samples was conducted by visualizing the results acquired from the R package VennDiagram [61]. Functional annotations of DEGs were conducted using MapMan Mercator4 V2.0 [62] and the Plant Metabolic Pathway Databases (https://plantcyc.org/ (accessed on 17 March 2024)). Information about genes involved in hormone synthesis was also supplemented by the Plant Hormone Gene Database (PHGD) (http://bioinfo.cemps.ac.cn/GSHR/ (accessed on 10 May 2024)) [63].

The weighted gene co-expression network analysis (WGCNA) was conducted utilizing version 1.47 of the WGCNA package within R [64]. This analysis identified gene modules, or clusters of co-expressed genes, which may indicate shared regulatory mechanisms. The input for the WGCNA analysis consists of DEGs identified between any two time points and across two treatments. To ensure the gene network conformed to a scale-free topology, a β value was selected to create an adjacency matrix. In this context, the soft threshold (or power) determines the weighting, while the *y*-axis reflects the relationship between node connectivity kk and its frequency p(k)p(k). A satisfactory scale-free model typically requires a correlation between kk and p(k)p(k) of at least 0.85. Following the WGCNA user manual, various β shrinkage parameters were evaluated, and a β value of 8 was found to best fit the scale-free topology. Modules sharing a high degree of similarity, indicated by a correlation coefficient greater than 0.8 (equivalent to a dissimilarity coefficient less than 0.2), were merged.

The gene ontology (GO) annotations for the proteins were completed using the eggNOG-mapper v2 (http://eggnog6.embl.de/#/app/emapper (accessed on 5 June 2023)) [65] and KEGG pathways performed through the KEGG Automatic Annotation Server (KAAS) [66]. After building the R package OrgDB of using the R package AnnotationForge [67], GO and KEGG pathway enrichment analysis of DEGs was implemented using the R package clusterProfiler [68], with padj < 0.05 considered as the threshold. Full-length protein sequences were queried against iTAK v1.5 [69] to acquire the composition of each TF family in the wheat genome, to which different DEG groups were aligned.

The gene co-expression analysis pipeline harnesses the capabilities of tidyverse and an advanced graph analytical method called Simple Tidy GeneCoEx [70]. Within the R environment, a comprehensive workflow was implemented utilizing several R packages. Initially, Z-score normalization (z = (x-mean)/sd) was conducted on the gene transcriptome data to ensure appropriate scaling and centering. To construct a gene interaction network based on significant correlations, Pearson correlation coefficients between genes were first calculated using the cor() function. Subsequently, for each pair of genes with a positive correlation (*r* > 0), the t-statistic was computed using the formula:

$$t=r\sqrt{\frac{n-2}{1-r^{2}}}$$

where *n* is the number of samples. The corresponding *p*-values were derived from the t-distribution. To control for multiple testing, false discovery rate (FDR) correction was applied to the *p*-values. Gene pairs that satisfied both conditions of positive correlation (*r* > 0) and an FDR-adjusted *p*-value less than 0.01 were retained as edges in the interaction network.

To delineate modules related to bait genes, the Leiden community detection algorithm was employed via the cluster_leiden() function from the igraph package. The identified modules were then visualized using the ggplot2 and ggraph packages, providing insightful graphical representations of the co-expression networks.

### 4.5. Validation of Transcriptome Results Using Quantitative Real-Time PCR

To validate the transcriptome sequencing (RNA-Seq) results, quantitative real-time PCR (qPCR) was performed on a 7500 Fast Real-Time PCR System (Applied Biosystems, Foster City, CA, USA). The reactions were carried out using ChamQ SYBR qPCR Master Mix (Vazyme, Nanjing, China) following this protocol: an initial denaturation at 95 °C for 30 s, followed by 40 cycles of 95 °C for 10 s and 60 °C for 30 s. A melting curve analysis was conducted with a ramp from 60 °C to 95 °C, holding for 1 min at 60 °C and concluding with 30 s at 95 °C. For normalization, TaActin, which exhibits stable expression across samples, served as the internal control. The relative quantification of gene expression was calculated using the 2−ΔΔCT method. Each experimental condition included three biological replicates, each analyzed in triplicate to ensure data reliability. The gene-specific primer sequences used in this study are provided in Supplementary Table S5.

## 5. Conclusions

Our study explored the effects of different light conditions, with a focus on blue light and on the growth, development, and transcriptomic responses of wheat plants. Blue light exposure resulted in reduced plant stature, accelerated spike development, and a shortened flowering period. A systematic investigation of transcriptome dynamics under varying light conditions uncovered key transcription factors and genes. This enhances our understanding of the mechanisms by which light quality influences morphogenesis, architecture, and flowering in wheat, with potential implications for the development of LED-mediated light quality control to optimize the growth and development of plants in greenhouses.

## Figures and Tables

**Table 1:** Plant growth and development under different light qualities (mean ± standard error [S.E.]).

Treatment	Survival Rate (%)	Heading Date (d)	Biomass Yield on the Ground (g)
White	90 ^a^ ± 2.1	62.0 ^a^ ± 2.8	1.31 ^a^ ± 0.04
Blue	93.3 ^a^ ± 1.6	48.5 ^b^ ± 2.7	1.26 ^a^ ± 0.03

Note: different lowercase letters indicate significant differences at the *p* < 0.05 level, determined by the *t*-test.

**Table 2:** Plant growth and development under different blue spectral intensities (mean ± S.E.).

EQ (μmol/m^2^ s)	Survival Rate (%)	Heading Date (d)	Weight of Dry Matter on the Ground (g)
56.7	90.0 ^ab^ ± 1.3	52.32 ^a^ ± 1.1	1.21 ^a^ ± 0.03
49.3	93.3 ^a^ ± 2.6	53.08 ^ab^ ± 0.6	1.17 ^ab^ ± 0.03
35.6	83.0 ^b^ ± 2.9	54.50 ^bc^ ± 1.0	1.11 ^b^ ± 0.02
23.7	24.0 ^c^ ± 1.6	56.83 ^c^ ± 2.10	0.84 ^c^ ± 0.05

Note: different lowercase letters indicate significant differences at the *p* < 0.05 level, determined by one-way ANOVA followed by Tukey’s HSD test.

**Table 3:** Illumination parameters of different light treatments.

Light	PPFD (μmol/m^2^ s)	Ep (Wphyto/m^2^)	Ef (W/m^2^)	Ratio_R (%)	Ratio_G (%)	Ratio_B (%)
Mix (R:B1:1)	180.53	32.693	0.0047234	55.3	1.8	41.9
White	182.1	33.225	1.7378	15.7	80.6	3.7
Red	185.81	31.962	0.042989	99.3	0.7	0
Blue	182.19	35.167	0.0028182	0	5	95

## Data Availability

The raw data of RNA-seq was uploaded to the NCBI under the accession number PRJNA1170217. All data supporting this research result can be obtained in the paper and within its Supplementary Materials published online.

## Supplementary Materials

The following supporting information can be downloaded at: https://www.mdpi.com/article/10.3390/plants14010046/s1, Figure S1: Distribution of DEG Numbers Across Light Conditions and Time Points; Figure S2: Expression Patterns of Genes Categorized by Masigpro; Figure S3: GO Enrichment Analysis of DEGs at Various Time Points Under Different Light Conditions; Figure S4: WGCNA Analysis; Figure S5: Simple Tidy GeneCox analysis; Figure S6: Expression Pattern of TaCRY; Table S1: Light Condition; Table S2: Sequencing Quality Information. Table S3: Distribution of Transcription Factors in Differential Genes. Table S4: Enrichment Results of GO in WGCNA Modules. Table S5: Primers for qRT-PCR. Table S6: Homology Analysis of 18 Wheat Transcription Factors Across Species.
