# Supplementary material for: Transcriptomic Analysis of Wheat Under Multi LED Light Conditions
Source: Plants (Basel). 2024 Dec 27;14(1):46. doi: 10.3390/plants14010046 (PMC11723344; doi:10.3390/plants14010046)
Supplement: Supplementary file 1 [file plants-14-00046-s001.zip › Figure S1. Distribution of DEG Numbers Across Light Conditions and Time Points.pdf]

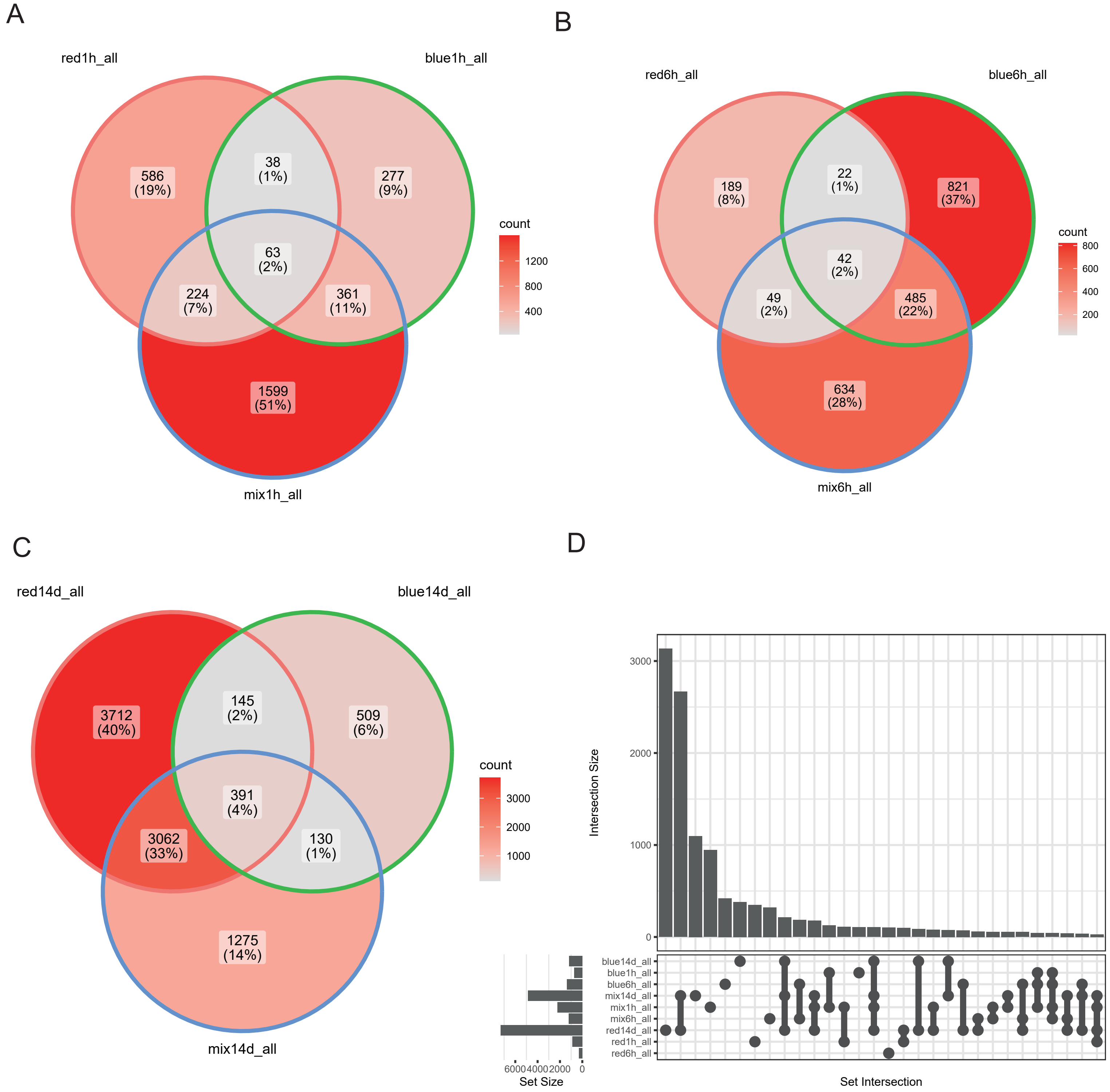

Figure S1: Distribution of DEG Numbers Across Light Conditions and Time Points

A-C. Venn Diagrams: DEGs comparing red, blue, combined red-blue, and white light at 1h (A), 6h (B), and 14d (C).

D. UpSet Plot: Summary of DEG intersections across all time points and light conditions.
