# Supplementary material for: Transcriptomic Analysis of Wheat Under Multi LED Light Conditions
Source: Plants (Basel). 2024 Dec 27;14(1):46. doi: 10.3390/plants14010046 (PMC11723344; doi:10.3390/plants14010046)
Supplement: Supplementary file 1 [file plants-14-00046-s001.zip › Figure S3. GO Enrichment Analysis of DEGs at Various Time Points Under Different Light Conditions.pdf]

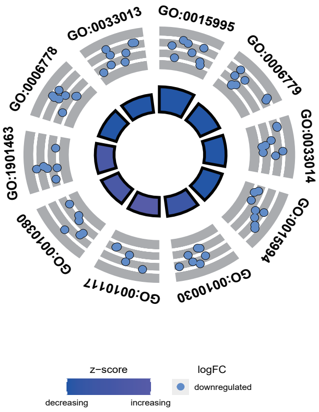

ID	Description
GO:0015995	chlorophyll biosynthetic process
GO:0006779	porphyrin−containing compound biosynthetic process
GO:0033014	tetrapyrrole biosynthetic process
GO:0015994	chlorophyll metabolic process
GO:0010030	positive regulation of seed germination
GO:0010117	photoprotection
GO:0010380	regulation of chlorophyll biosynthetic process
GO:1901463	regulation of tetrapyrrole biosynthetic process
GO:0006778	porphyrin−containing compound metabolic process
GO:0033013	tetrapyrrole metabolic process

1h_white_vs_blue light

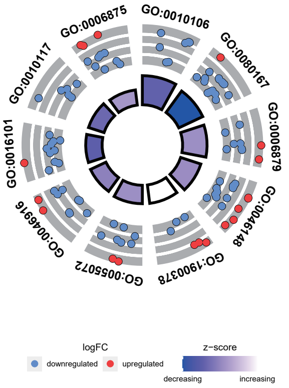

ID	Description
GO:0010106	cellular response to iron ion starvation
GO:0080167	response to karrikin
GO:0006879	cellular iron ion homeostasis
GO:0046148	pigment biosynthetic process
GO:1900378	positive regulation of secondary metabolite biosynthetic process
GO:0055072	iron ion homeostasis
GO:0046916	cellular transition metal ion homeostasis
GO:0016101	diterpenoid metabolic process
GO:0010117	photoprotection
GO:0006875	cellular metal ion homeostasis

6h_white_vs_blue light

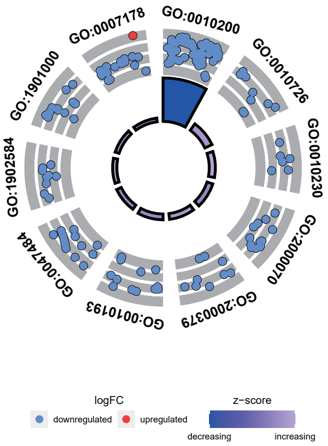

ID	Description
GO:0010200	response to chitin
GO:0010726	positive regulation of hydrogen peroxide metabolic process
GO:0010230	alternative respiration
GO:2000070	regulation of response to water deprivation
GO:2000379	positive regulation of reactive oxygen species metabolic process
GO:0010193	response to ozone
GO:0047484	regulation of response to osmotic stress
GO:1902584	positive regulation of response to water deprivation
GO:1901000	regulation of response to salt stress
GO:0007178	transmembrane receptor protein serine/threonine kinase signaling path

1h_white_vs_mix light

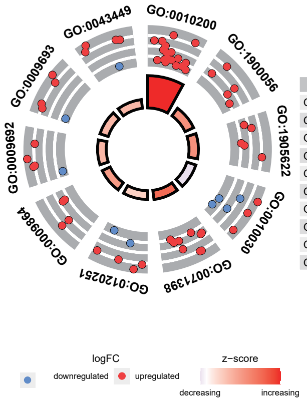

ID	Description
GO:0010200	response to chitin
GO:1900056	negative regulation of leaf senescence
GO:1905622	negative regulation of leaf development
GO:0010030	positive regulation of seed germination
GO:0071398	cellular response to fatty acid
GO:0120251	hydrocarbon biosynthetic process
GO:0009864	induced systemic resistance, jasmonic acid mediated signaling pathw
GO:0009692	ethylene metabolic process
GO:0009693	ethylene biosynthetic process
GO:0043449	cellular alkene metabolic process

6h_white_vs_mix light

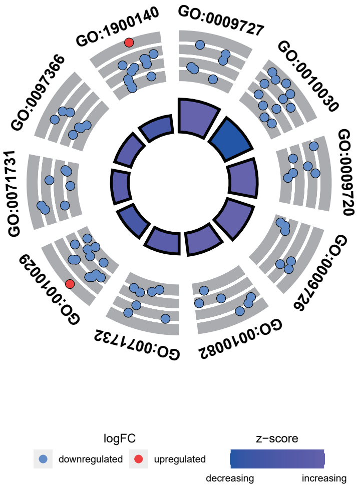

ID	Description
GO:0009727	detection of ethylene stimulus
GO:0010030	positive regulation of seed germination
GO:0009720	detection of hormone stimulus
GO:0009726	detection of endogenous stimulus
GO:0010082	regulation of root meristem growth
GO:0071732	cellular response to nitric oxide
GO:0010029	regulation of seed germination
GO:0071731	response to nitric oxide
GO:0097366	response to bronchodilator
GO:1900140	regulation of seedling development

14d_white_vs_mix light

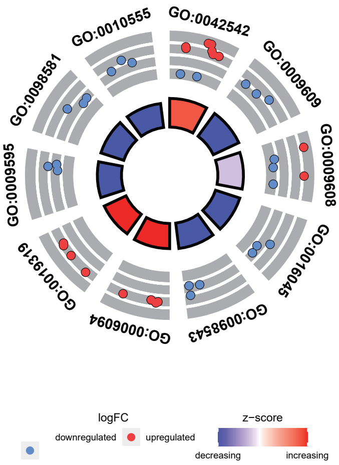

ID	Description
GO:0042542	response to hydrogen peroxide
GO:0009609	response to symbiotic bacterium
GO:0009608	response to symbiont
GO:0016045	detection of bacterium
GO:0098543	detection of other organism
GO:0006094	gluconeogenesis
GO:0019319	hexose biosynthetic process
GO:0009595	detection of biotic stimulus
GO:0098581	detection of external biotic stimulus
GO:0010555	response to mannitol

1h_white_vs_red light

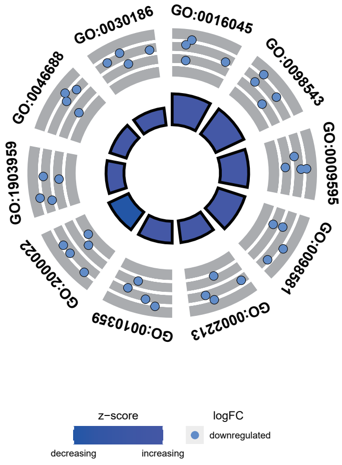

ID	Description
GO:0016045	detection of bacterium
GO:0098543	detection of other organism
GO:0009595	detection of biotic stimulus
GO:0098581	detection of external biotic stimulus
GO:0002213	defense response to insect
GO:0010359	regulation of anion channel activity
GO:2000022	regulation of jasmonic acid mediated signaling pathway
GO:1903959	regulation of anion transmembrane transport
GO:0046688	response to copper ion
GO:0030186	melatonin metabolic process

6h_white_vs_red light
