# Supplementary material for: Transcriptomic Analysis of Wheat Under Multi LED Light Conditions
Source: Plants (Basel). 2024 Dec 27;14(1):46. doi: 10.3390/plants14010046 (PMC11723344; doi:10.3390/plants14010046)
Supplement: Supplementary file 1 [file plants-14-00046-s001.zip › Figure S5. Simple Tidy GeneCox analysis.pdf]

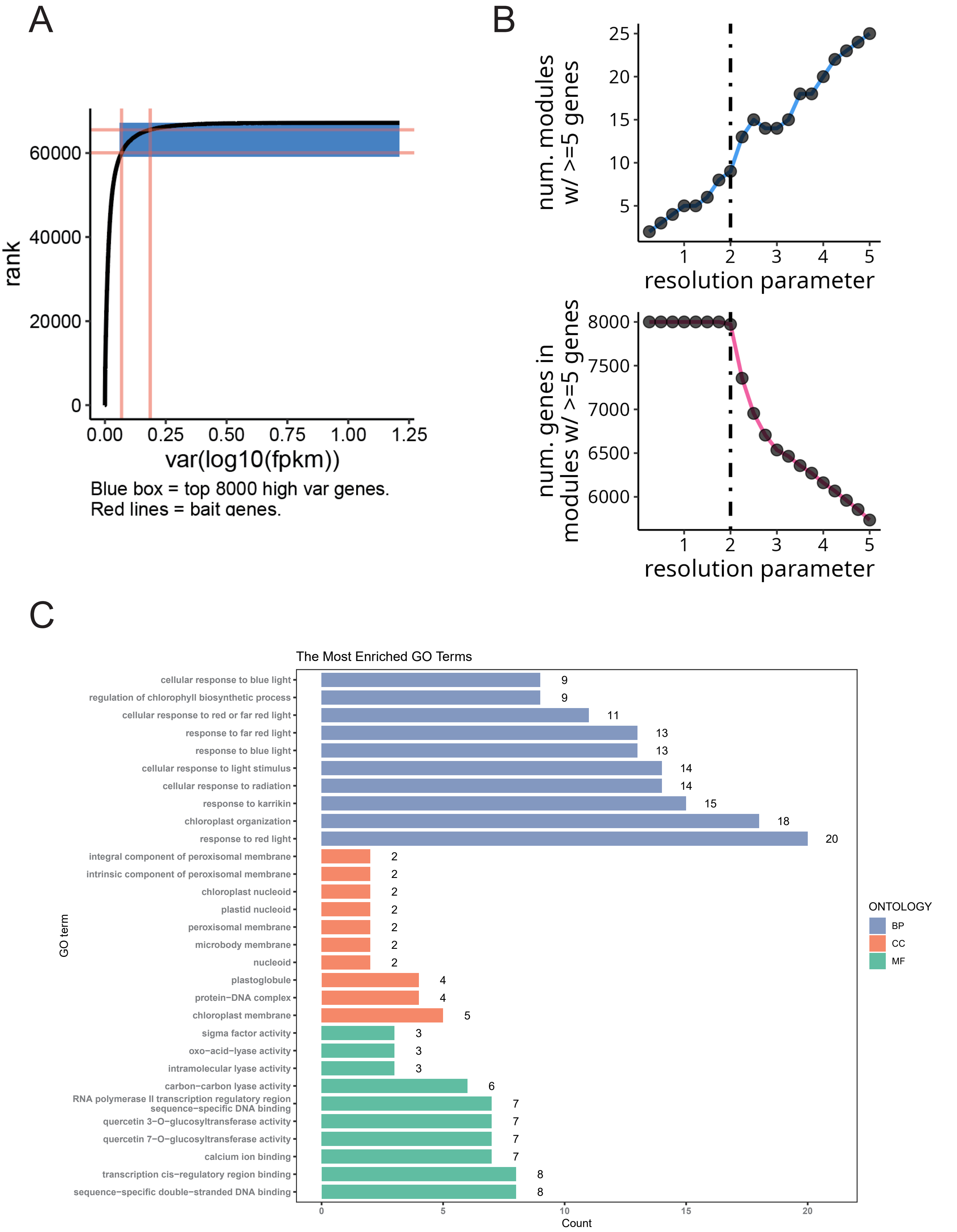

A. Distribution of high-variance genes. B.Resolution parameter selection.
C. GO enrichment analysis of 404 neighbor genes
