# Supplementary figures and images for: Transcriptomic Analysis of Wheat Under Multi LED Light Conditions
Source: Plants (Basel). 2024 Dec 27;14(1):46. doi: 10.3390/plants14010046 (PMC11723344; doi:10.3390/plants14010046)

Cluster 1

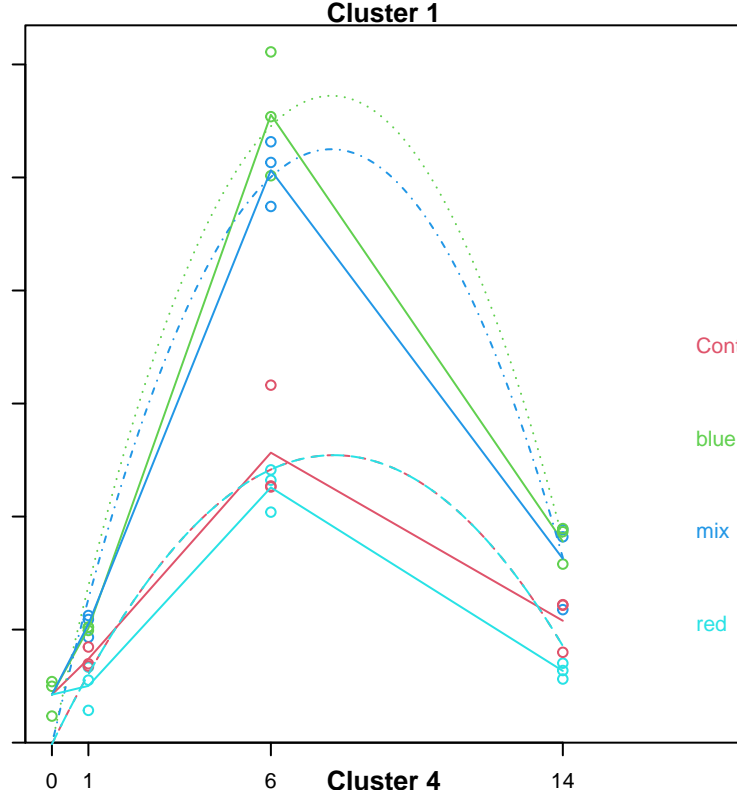

Cluster 2

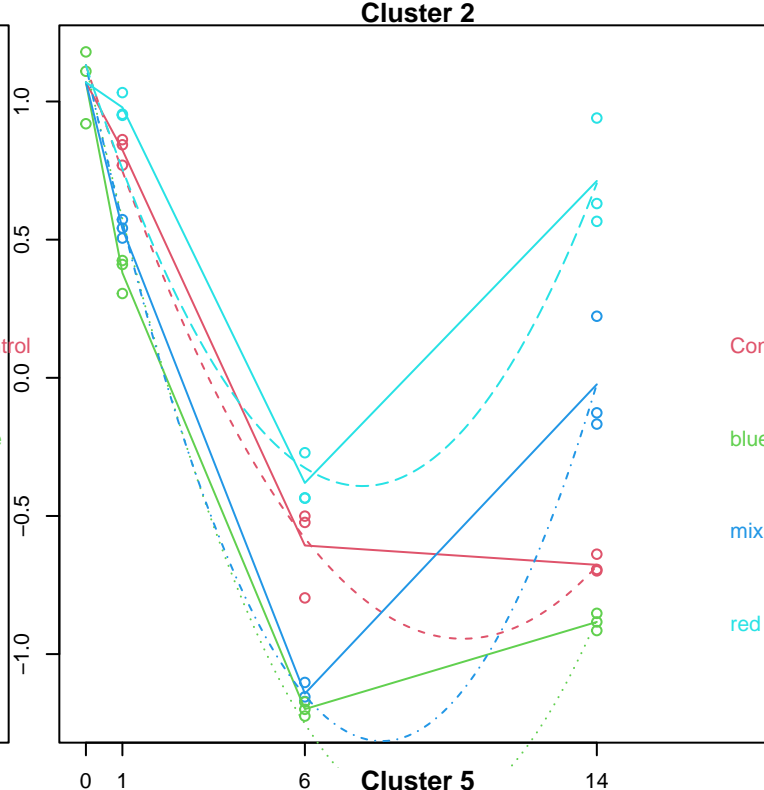

Cluster 3

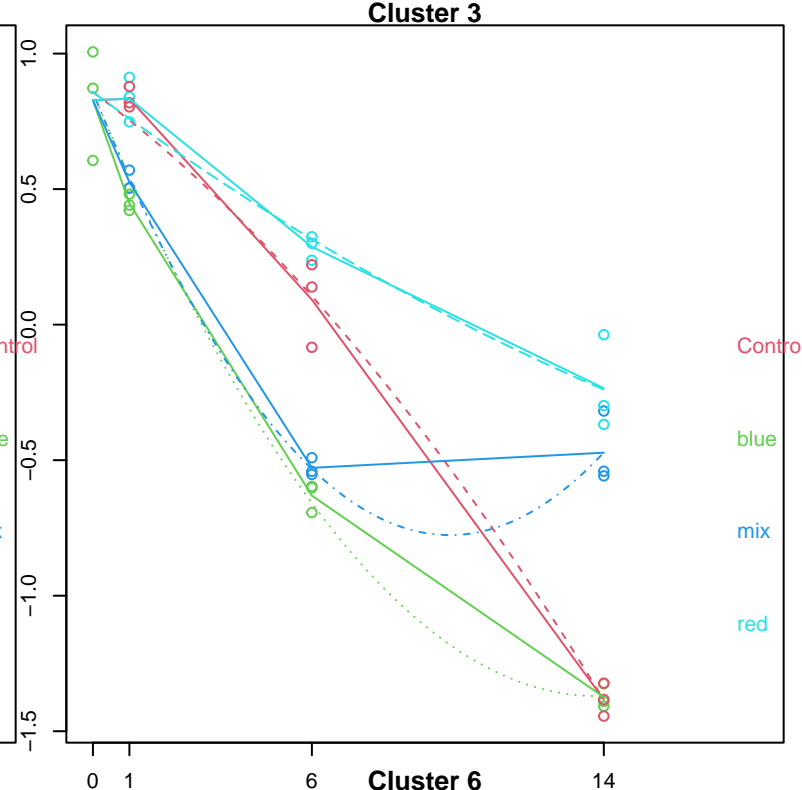

Cluster 4

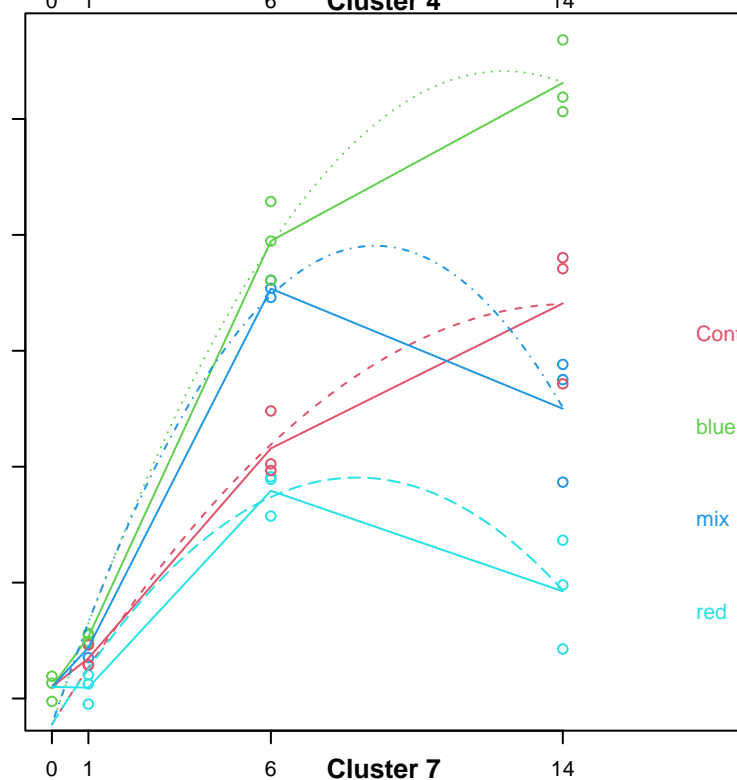

Cluster 5

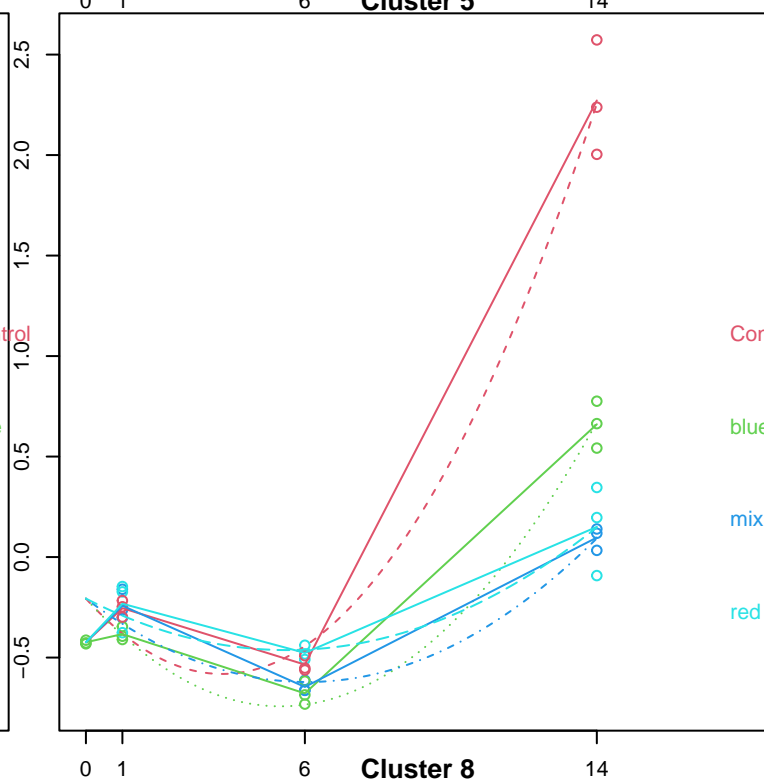

Cluster 6

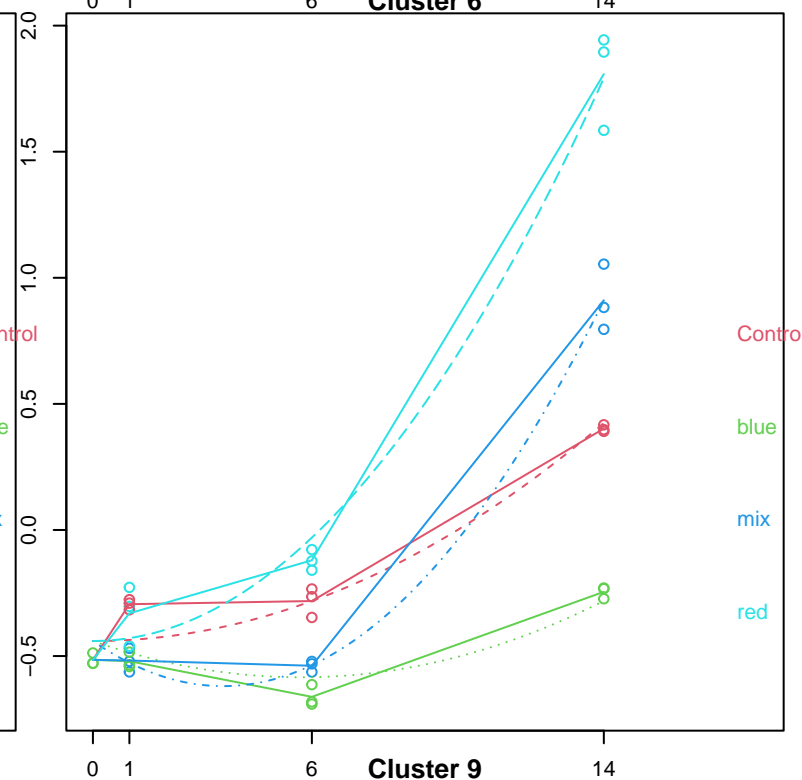

Cluster 7

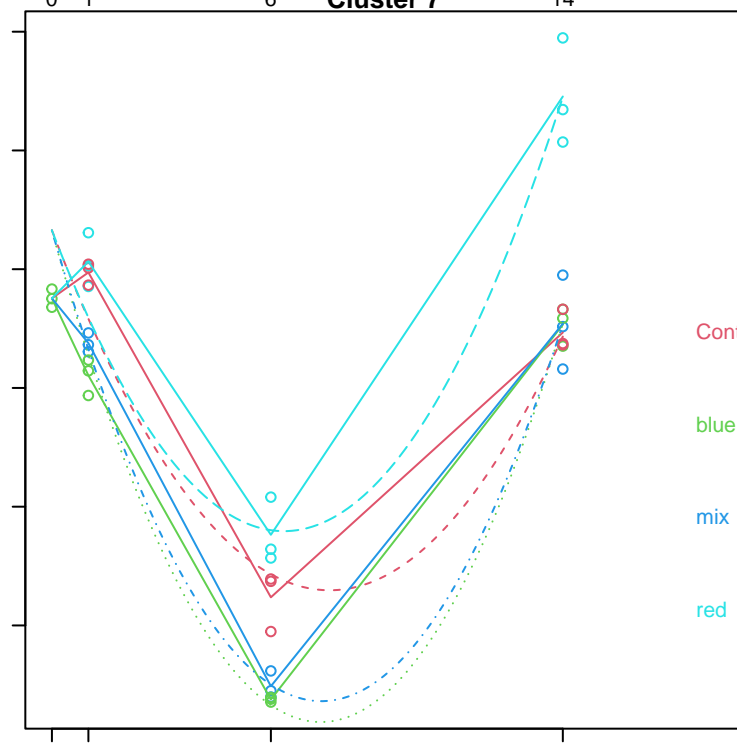

Cluster 8

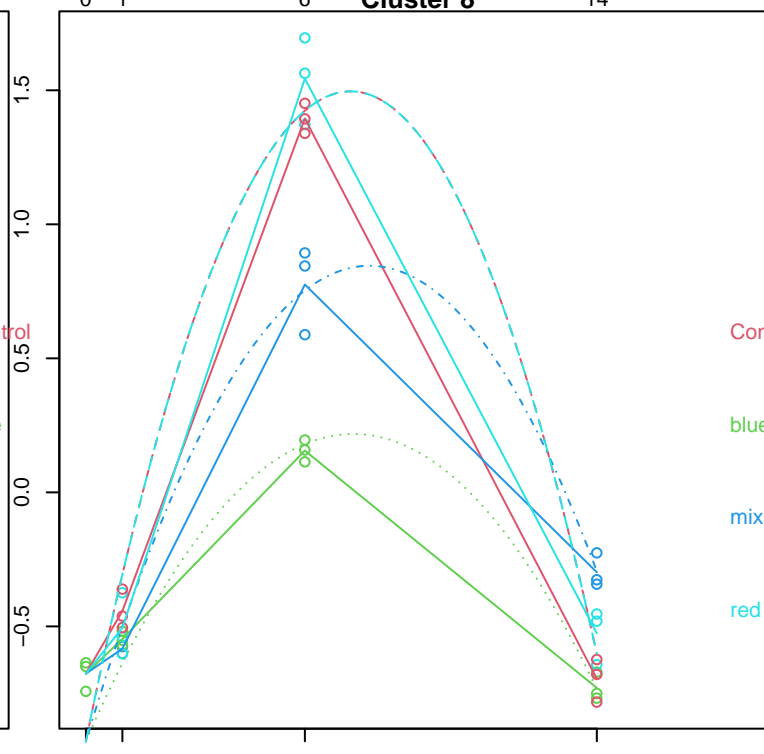

Cluster 9

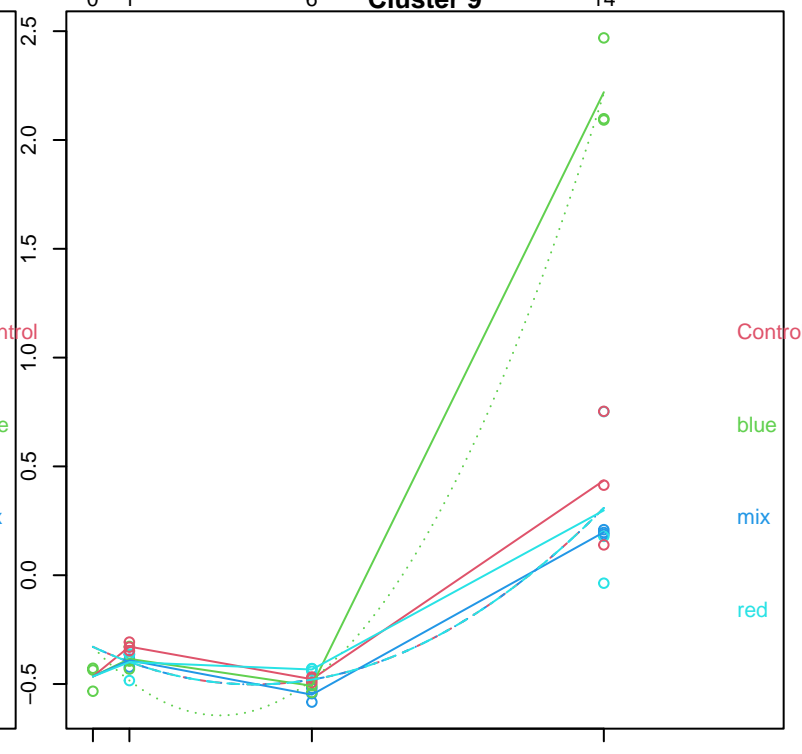

Supplement: Supplementary file 1 [file plants-14-00046-s001.zip › Figure S2. Expression Patterns of Genes Categorized by Masigpro.pdf]

A

Module-Sample relationships

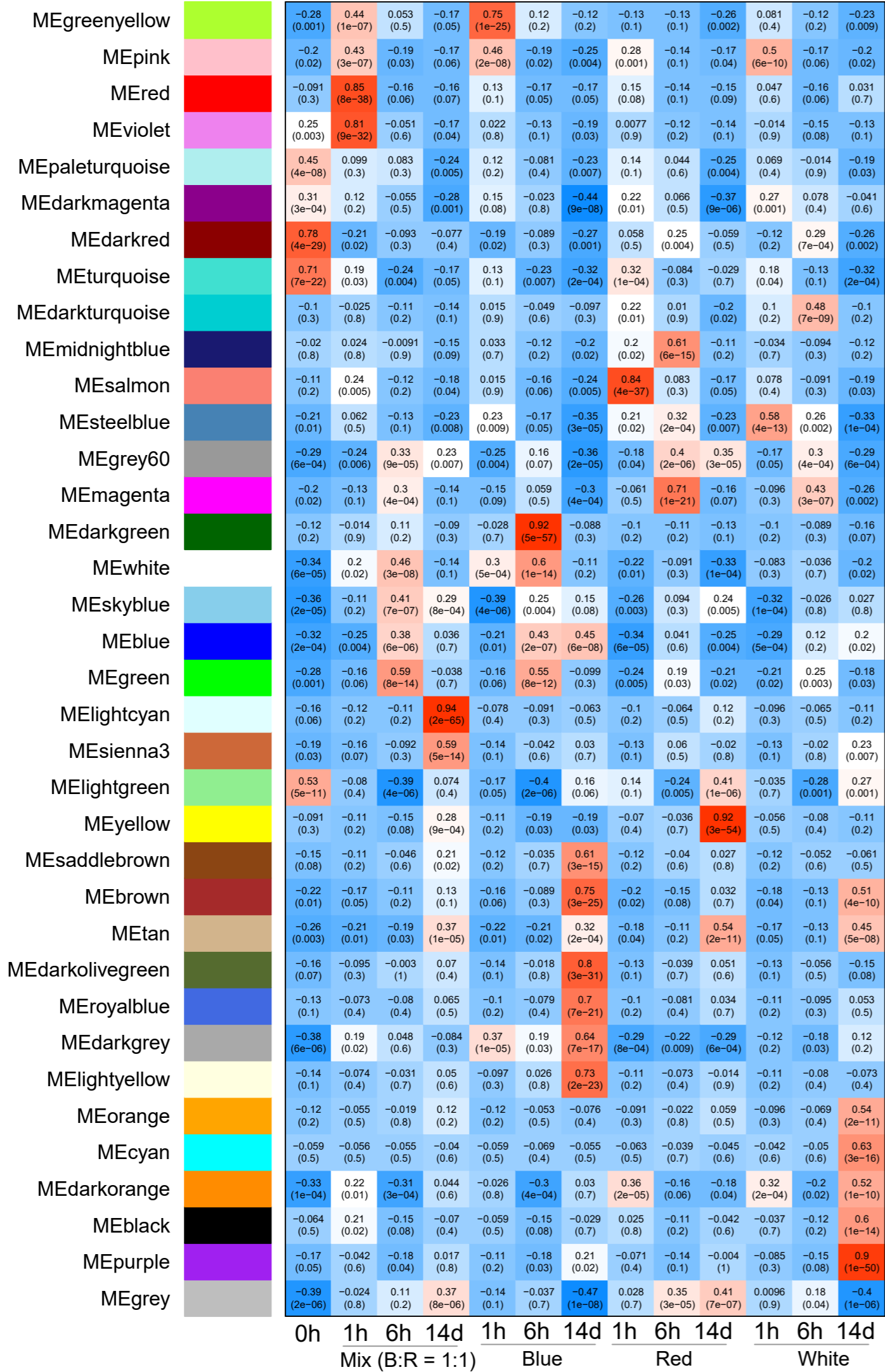

B

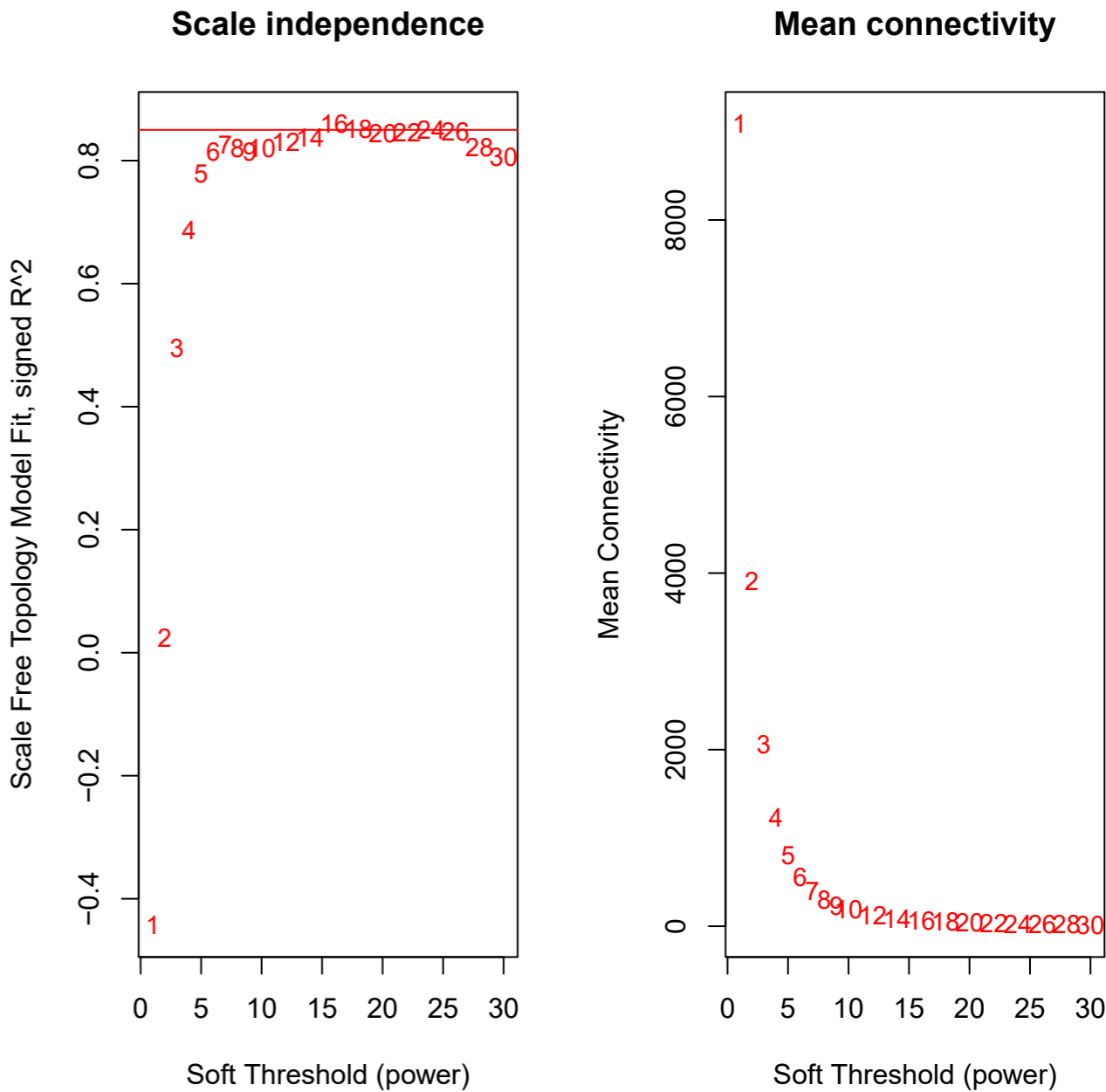

A.Module-Sample Relationships;B. Soft Threshold Selection

Supplement: Supplementary file 1 [file plants-14-00046-s001.zip › Figure S4. WGCNA analysis.pdf]

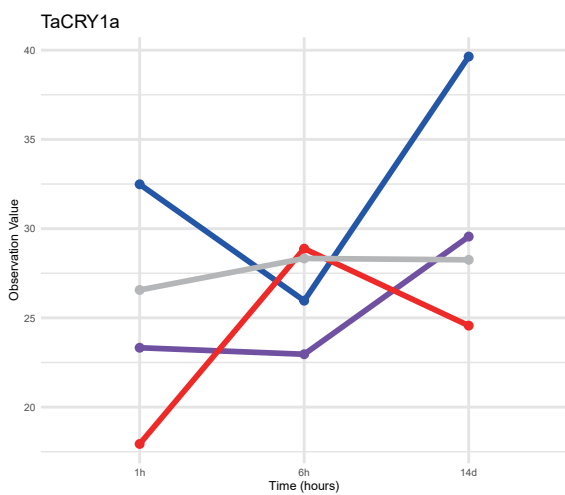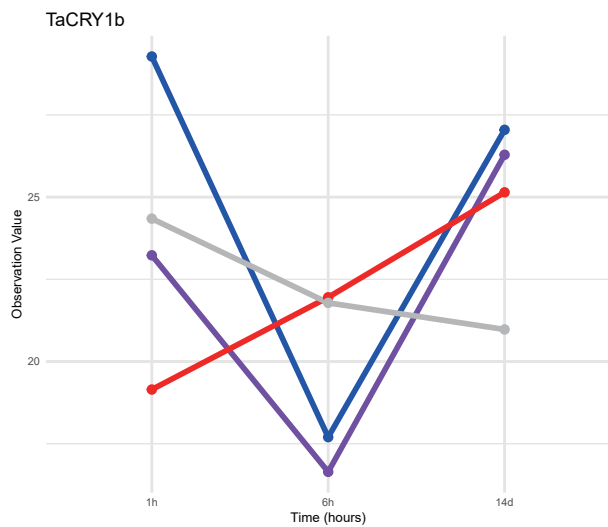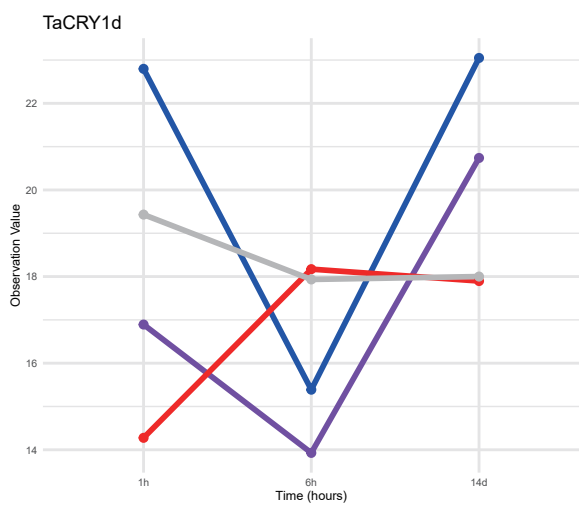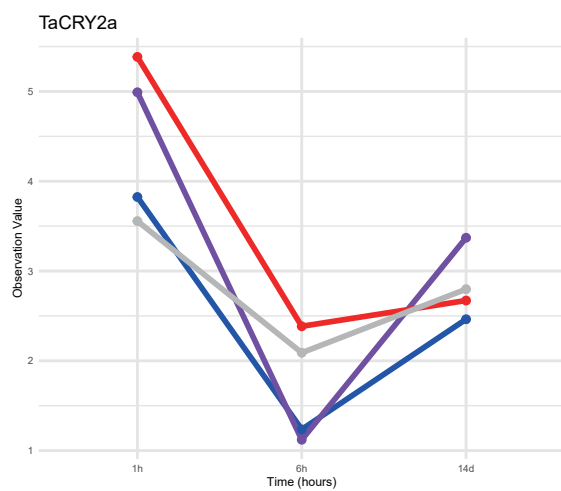

Treatment

blue

mix

red

white

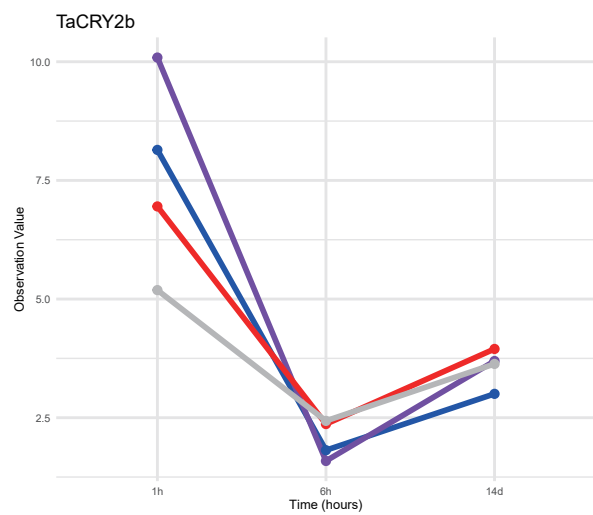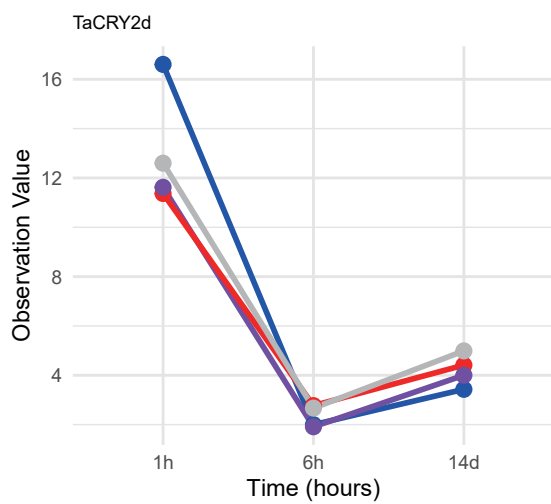

Supplement: Supplementary file 1 [file plants-14-00046-s001.zip › Figure S6. Expression Pattern of TaCRY.pdf]
